# Exogenous Melatonin Directly and Indirectly Influences Sheep Oocytes

**DOI:** 10.3389/fvets.2022.903195

**Published:** 2022-05-26

**Authors:** Yang Chen, Xuesong Shan, Huaizhi Jiang, Zhenhua Guo

**Affiliations:** ^1^Key Laboratory of Livestock and Poultry Resources (Sheep & Goat) Evaluation and Utilization, Ministry of Agriculture and Rural Affairs, College of Animal Science and Technology, Jilin Agricultural University, Changchun, China; ^2^Heilongjiang Academy of Agricultural Sciences, Animal Husbandry Research Institute, Harbin, China

**Keywords:** animal welfare, ewe, follicle, meta-analysis, development

## Abstract

Understanding whether and how melatonin (MT) may impact sheep oocyte development competence is central to our ability to predict how sheep oocytes will respond to artificially regulated estrus. Implanting MT can make sheep enter estrus during the non-breeding season. One study found that the blastocyst rate increased under MT treatment, while another found that the blastocyst rate decreased. Therefore, we conducted a meta-analysis of MT directly and indirectly influencing sheep oocytes. A total of 433 articles were collected from which 20 articles and 34 treatments were finally selected. A method for estimating the default value was established for the litter size analysis. We found that exogenous MT add into *in vitro* maturation medium was positively related to the blastocyst rate in the lab. However, subcutaneous implanting MT did not affect the *in vivo* ovulation rate, fertilization rate, blastocyst rate, or pregnancy rate at farm. MT did not affect the *in vitro* cleavage rate. However, MT improved the *in vivo* cleavage rate. We hypothesized that implanted MT could increase the concentration of MT in oviduct fluid *in vivo*, and also that *in vitro* MT could increase the early cleavage rate of sheep zygotes without affecting the total cleavage rate. In the analysis of oocyte apoptosis caused by injury, the results suggested that pyroptosis would be more suitable for further research. MT produces responses in all body organs, and thus implanting of MT during non-breeding seasons should consider the effect on animal welfare.

## Introduction

Sheep oocyte development competence can be directly affected by adding exogenous melatonin (MT) during *in vivo* culture as well as indirectly by implanting MT in ewes. The concentration of MT in various tissues differs widely. The concentration of MT in the gastrointestinal tract is 10–100 times higher than that in blood (1). The concentration of MT is different in different size sheep follicles (2). This indicates that MT is positively correlated with the developmental ability of sheep oocytes. MT can maintain the reproductive process (3). MT has anti-inflammatory (4) and wound healing functions (5) and is associated with many signaling pathways (6). This article focuses on the impact of MT on sheep oocytes. Different drug delivery routes can have different effects; for example, magnesium sulfate is taken orally for catharsis (7), and intramuscular or intravenous injection can relieve muscle spasms (8). Therefore, we were curious to know how MT directly and indirectly affects sheep oocytes.

Sheep are seasonal estrous animals. The estrous period and intensity are significantly different depending on the region, breed, and other factors (9). Implanting MT can make sheep enter estrus in non-breeding seasons. However, this phenomenon is not uniform in all aspects. One study found that blastocyst cell number decreased (10), whereas another study found that blastocyst cell number increased (11). Some studies have suggested that the blastocyst rate is decreased (12), whereas others have found that the blastocyst rate is increased (13). In particular, litter size changes with the season (9, 14). According *in vitro* study, the blastocyst rate of cultured sheep oocytes can be improved by adding MT to *in vitro* maturation (IVM) (10, 11, 15). However, there is no report on the simultaneous control study of *in vivo* and *in vitro* studies. Therefore, we performed a meta-analysis and reviewed the results concerning the direct and indirect effects of MT on sheep oocytes.

## Methods

### Database Search Strategy

We collected articles concerning the effects of MT on sheep oocytes both *in vitro* and *in vivo*. A search of PubMed, Ovid, Science Direct, and ProQuest from 2006.01.01 to 2021.09.01 used the key search terms melatonin AND (Ovine OR sheep OR ewe) AND (oocyte OR follicle), producing a total of 238 articles. The effects of melatonin on litter size used the key search terms melatonin AND (ram OR ewe) AND implant, and 195 articles were found.

### Data Extraction

We selected the relevant articles according to the criteria listed in Table 1. Specifically, oocytes cultured *in vitro* should have follicle growth data or embryonic development data. In the experiment of subcutaneous implantation in ewes, ovulation data or oocyte development data should be available. The data of litter size should be available in the experiment of ram subcutaneous implantation. The studies of melatonin's effect on sheep oocyte development tended to explore the mechanism, and many experimental groups had observed positive results. When extracting the data, only the groups with the largest positive value were retained. Each experimental group was studied in different seasons or in different sheep varieties about litter size study. Therefore, when extracting data, each experimental group was used as a treatment. When the experimental data were incomplete, we contacted the authors to obtain the data. We were unable to contact all the authors to obtain the raw data. When analyzing the effect on litter size, we supplemented the missing data using the default value; refer Supplementary Data 1 for the method.

**Table 1:** Inclusion and exclusion criteria.

**Inclusion**	**Exclusion**
Species evaluated included but was not limited to sheep	Sheep were not used
English literature	Non-English
Melatonin treatment alone or with other treatments in sheep	No melatonin treatment of sheep
Oocyte or reproduction data included	No oocyte or reproduction data

### Data Analysis

Review Manager (Copenhagen: Nordic Cochrane Center, Cochrane Collaboration, Version 5.4) was used for the meta-analysis. A standard mean difference (SMD) meta-analysis using a continuous model was used to calculate *in vitro* culture follicle diameter and blastocyst cell number. The effects of implanting MT on ovulation rate, fertilization rate, and litter size were also analyzed with a continuous model. Relative risk (RR) was analyzed with a dichotomous model. This included *in vitro* culture follicle normal rate, IVM rate, cleavage rate, and blastocyst rate. The effects of implanting MT on cleavage rate, blastocyst rate, and pregnancy rate were also analyzed with a dichotomous model.

Heterogeneity was analyzed by the Higgins *I*^2^ statistic (*I*^2^ >50% means a high level of heterogeneity) (16). When heterogeneity was found in the analysis, the source of the heterogeneity was identified according to the differences in follicle diameter and culture methods as well as the experimental details such as different research seasons (Table 2). The heterogeneous groups were analyzed by a random effects model. The groups without heterogeneity were analyzed by a fixed effects model. Litter size was analyzed without considering subgroups, as the location in the northern and southern hemispheres and different time periods of the studies were the sources of heterogeneity. Potential bias was identified using Begg's funnel plots and rechecked by Egger's linear regression (28) and Begg's rank correlation tests. Statistical analyses were performed using Stata 12.0 (Stata Corp, College Station, TX, USA). Furthermore, trial sequential analysis (TSA) was used to evaluate the reliability of our results. For the TSA we set the IVM rate control to 70% according to experimental experience.

**Table 2:** Characteristics of studies included.

	**Study**			**IVC**						
	**Year**	**Breed**	**Location and latitude**	**Treated**	**Oocytes recovered**	**Culture method**	**Culture Time (h)**	**Mature oocytes treated**	**Embryo IVC medium**	**Cleavage observed time**
1	Barros et al. (17)	Mixed-breed	Petrolina, Brazil S 16.1	Secondary follicles, >295 and <330 μm	IVC follicles	100 μl droplet	Follicle, 18 days IVM,32–48 h	NM	NM	NM
2	Barros et al. (18)	Mixed-breed	Petrolina, Brazil S 16.1	Early antral follicles, >400 and <500 μm	IVC follicles	100 μl droplet	Follicle, 12 days IVM, 36 h	NM	NM	NM
3	Deng et al. (19)*	NM	Beijing, China N 39.5	COCs	Abattoir	6-well plate	IVM, 19 h	Sperm injection	mSOF	120 h
4	Goodarzi et al. (11)	Lory Bakhtiary	Karaj, Iran N 35.8	COCs	Abattoir	50 μl droplet	IVM, 24 h	IVF	SOF	48 h
5	Tian et al. (15)	NM	Beijing, China N 39.5	COCs	Abattoir	60 μl droplet	IVM, 24 h	PA	mSOF	48 h
6	Tian et al. (10)	Merino	Beijing, China N 39.5	COCs	OPU	4-well plate	IVM, 24 h	IVF	SOF	48 h
				**Implant ewe**						
		**Breed**	**Location and latitude**	**Seasons**	**Oocytes recovered**	**Time, month**	**Live weight/age**	**Melatonin treatment**	**Embryo IVC medium**	**Cleavage observed time**
7	Buffoni et al. (20)	Merino	Trelew, Argentina S 43.0	Breeding and anestrous	OPU	Feb to Apr Sep to Nov	Adult, 63 kg 61 kg	58 days	NM	NM
8	Fang et al. (13)	Hu-sheep	Tianjin, China N 39.1	12 light:12 dark, in temperature control room	OPU	NM	4 week old	17 days	SOF	48 h
9	Vazquez, 2009	Rasa Aragonesa	Zaragoza, Spain N 41.4	breeding and anestrous	OPU	Jan Mar	64 kg 57 kg	42 days	SOF	NM
10	Vazquez et al. (21)	Rasa Aragonesa	Zaragoza, Spain N 41.4	breeding and anestrous	OPU	Jan Mar	64 kg 59 kg	42 days	SOF	24 and 36 h
11	Vazquez et al. (22)	Rasa Aragonesa	Zaragoza, Spain N 41.4	lactation	OPU	Feb Apr	61 kg	45 days	SOF	24 and 36 h
12	Vazquez et al. (12)	Rasa Aragonesa	Zaragoza, Spain N 41.4	parturition	OPU	Nov	61 kg	45 days	SOF	24 and 36 h
13	Abecia et al. (23)	Rasa Aragonesa	Spain N 41.4	Breeding	Ewe	Mar	56.3 kg	NM	41 days	40 days
					**Implant**	**Ram/ewe**	**For litter size**			
		**Breed**	**Location and latitude**	**Seasons**	**Treat**	**Time, month**	**Ewe Weight/age**	**Ram age**	**Melatonin treatment**	**Ram introduction**
14	Abecia et al. (9)	Rasa Aragonesa Assaf, Merino	Zaragoza Zamora Badajoz	NM	Ram Ewe	Jan, Feb, Apr, May	NM	NM	Ram 49 days Ewe 42 days	45 days
15	Cosso et al. (24)	Sarda	Sardinia, Italy N 40.5	breeding	Ram	Jun Jul	32 kg 240 days	2.5–6.5 years	35 days	45 days
16	Cosso et al. (24)	Romney composite	New Zealand N 40	Not breeding	Ewe	Oct	NM	NM	35 days	22 days
17	Luridiana et al. (25)	Sarda	Sardinia, Italy N 39.5	breeding	Ewe	Mar	3–6 years	NM	35 days	40 days
18	Mura et al. (26)	Sarda	Sardinia, Italy N 40.5	breeding	Ewe	Jun	26 kg 195 days	NM	35 days	40 days
19	Mura et al. (14)	Sarda	Sardinia, Italy N 40.0	NM	Ram Ewe	Feb, Mar Apr, May	3–6 years	NM	35 days	45 days
20	Mura et al. (27)	Sarda	Sardinia, Italy N 40.0	NM	Ram Ewe	Mar	3–5 years	At least 3 years	34 days	40 days

*Contacted the author to obtain experimental data.

*NM, Not mentioned; OPU, ovum pick-up; IVC, in vitro culture; IVM, in vitro maturation; IVF, in vitro fertilization; PA, parthenogenetic activation; SOF, synthetic oviduct fluid medium*.

## Results

**Figure 1:**
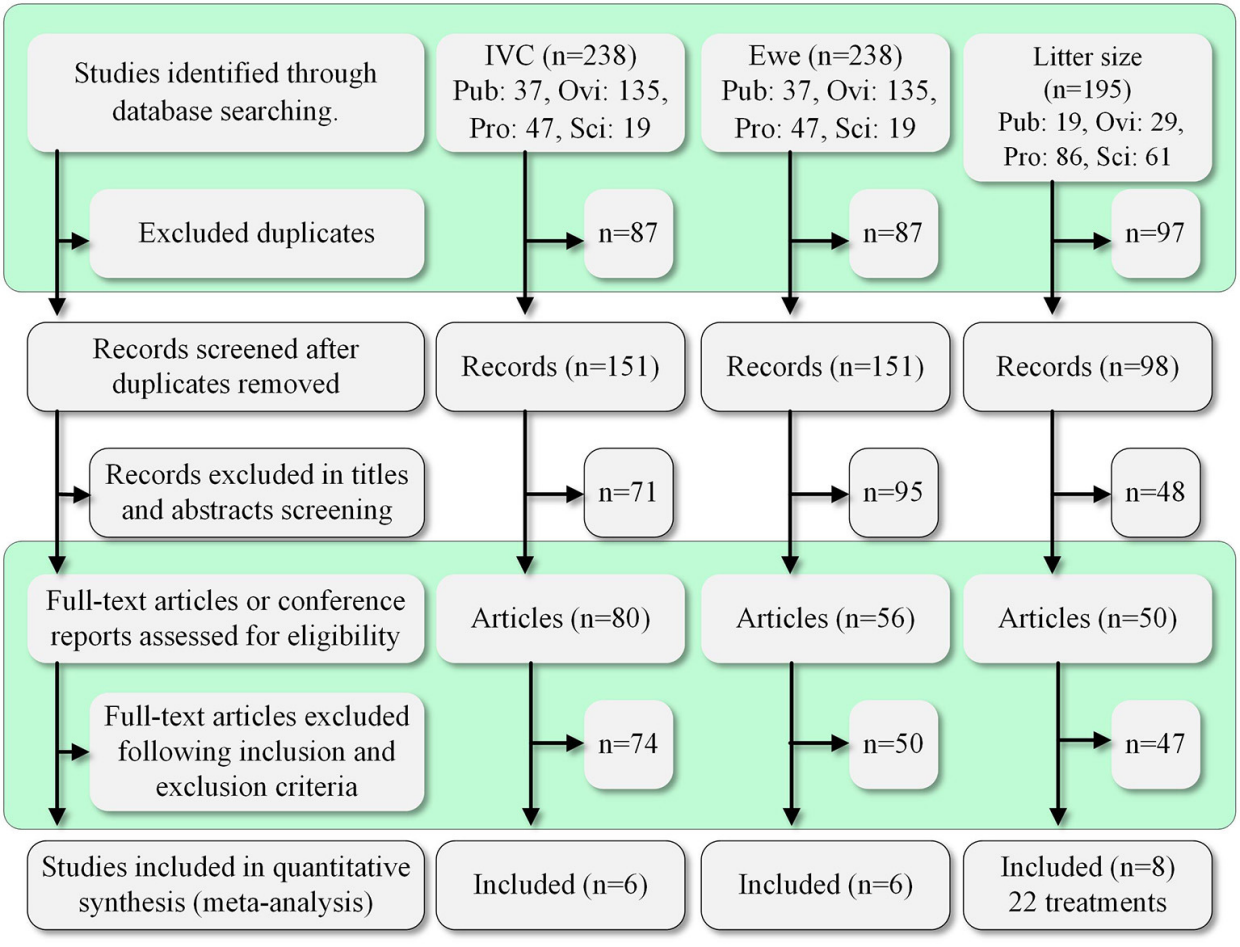
Summary of the study selection process. Pub, PubMed; Ovi, Ovid; Pro, ProQuest; Sci, ScienceDirect.

The process and results of screening 433 articles are shown in Figure 1. A final group of 20 studies is shown in Table 2 (9–15, 17–27, 29, 30). The studies comprised 34 treatments.

**Figure 2:**
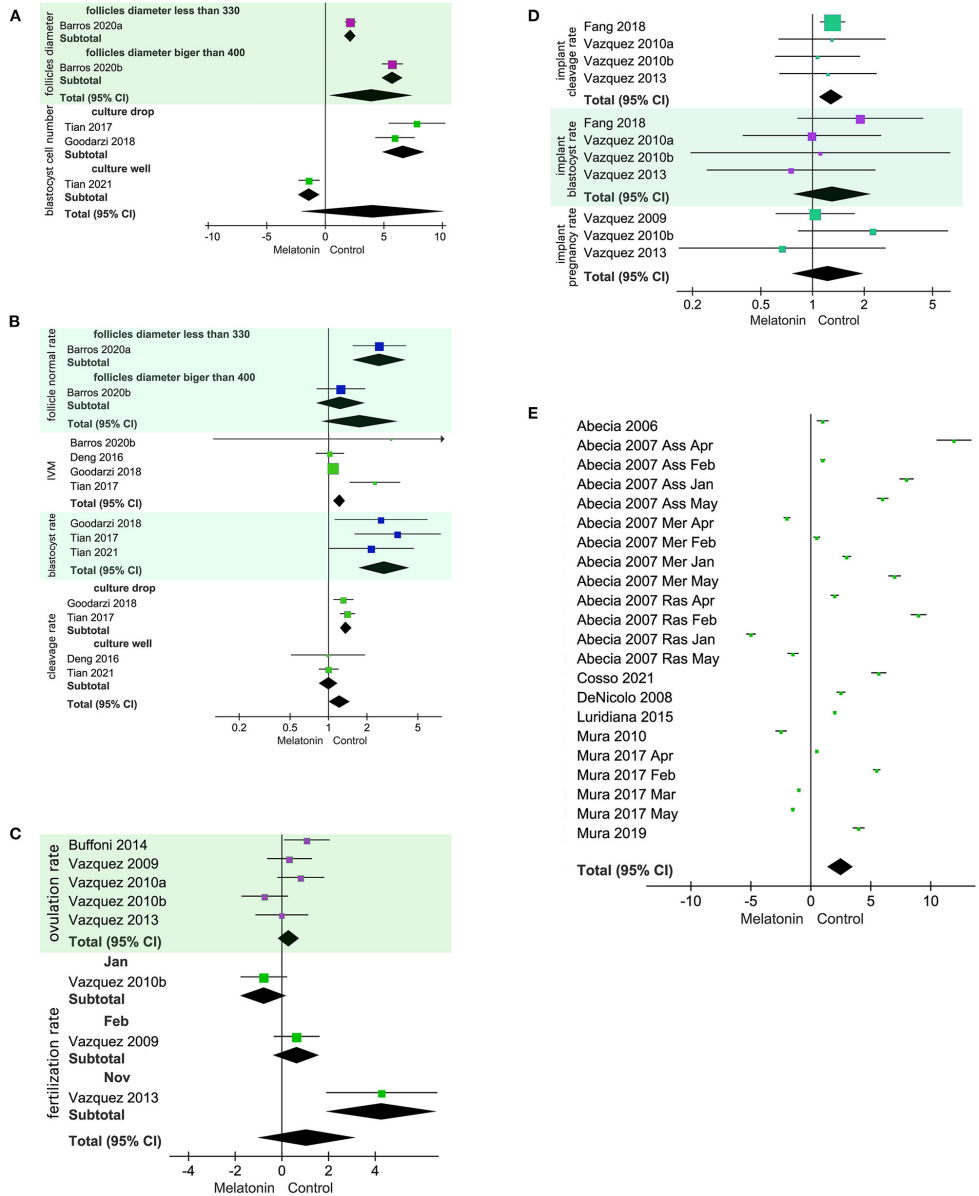
Forest plot of melatonin (MT) treatment effects on sheep oocytes. The subgroup analysis was based on different follicle diameters, different culture treatments, and different months. **(A)** SMD for the effects of *in vitro* culture of exogenous MT on follicle diameter and blastocyst cell number. Blastocyst cell number analysis culture droplet subgroup *I*^2^ = 36%. **(B)** RR for the effects of *in vitro* culture exogenous MT on follicle normal rate, IVM rate, cleavage rate, and blastocyst rate. Cleavage rate analysis of the culture droplet subgroup and the culture well subgroup yielded *I*^2^ = 0%. **(C)** SMD for implanted exogenous MT effects on ovulation rate and fertilization rate. **(D)** RR implanted exogenous MT effects on cleavage rate, blastocyst rate, and pregnancy rate. **(E)** Implanted exogenous MT effects on litter size.

Under *in vitro* conditions, exogenous MT was positively related to follicle diameter (SMD = 4.03, 95% CI = 0.42–7.63; *p* = 0.03; Figure 2A), *in vitro* maturation (IVM), and blastocyst rate (Figure 2B). MT had no effect on follicle normal rate (RR = 1.76, 95% CI = 0.89–3.47; *p* = 0.11; Figure 2B), cleavage rate (RR = 1.22, 95% CI = 1.01–1.47; *p* = 0.04; Figure 2B), or blastocyst cell number (SMD = 4.07, 95% CI = −2.08 to 10.22; *p* = 0.19; Figure 2A). There was significant heterogeneity in follicle diameter and follicle normal rate. Subgroup analysis according to follicle diameter was performed using a random model. The heterogeneity was produced by different follicle diameters. Cleavage rate and blastocyst cell number were heterogeneous owing to different culture methods according to the subgroup analysis using a random model. The sources of heterogeneity were culture wells and culture droplet size.

For *in vivo* studies using treated ewes, implanting MT had no effect on ovulation rate, fertilization rate (SMD = 1.08, 95% CI = −1.00 to 3.16; *p* = 0.31; Figure 2C), blastocyst rate, or pregnancy rate (RR = 1.29, 95% CI = 1.1–1.5; *p* = 0.56; Figure 2D). Implanting MT was positively related to cleavage rate (Figure 2D). The results for fertilization rate were heterogeneous due to differences in experimental starting month as decided by a subgroup analysis using a random model. When implanted MT treatment was used for rams or ewes, implanting MT was positively related to litter size (Figure 2E). The implanted MT litter size was 1.3395, and the control litter size was 1.2795.

**Figure 3:**
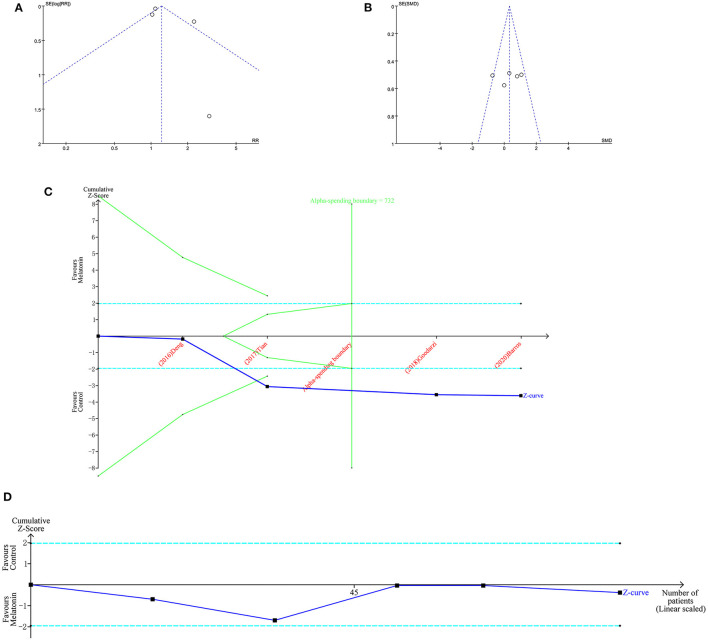
Bias analysis and TSA of MT of sheep oocytes. Funnel plots of IVM rate **(A)** and implant ovulation rate **(B)**. TSA of IVM rate **(C)** and implant ovulation rate **(D)**.

The funnel plot (Figures 3A,B) shows that there was no potential bias, and this result was corroborated by Egger's test (IVM rate, *p* = 0.972; implant ovulation rate *p* = 0.529) and Begg's test (IVM rate Pr > |*z*| = 1; implant ovulation rate, Pr > |*z*| = 0.602). The TSA results showed that the *in vitro* exogenous MT effect on sheep oocyte IVM was real (Figure 3C). It should be noted that the actual IVM rate was 80%−90%. We set the IVM rate control to 70%. Setting the control rate to 50% according to the statistical method would not allow the significance threshold to be reached. The influence of implanted MT on ewe oocytes needs further research (Figure 3D). Although the number of studies is large, the actual number of samples is small.

## Discussion

Understanding how MT directly and indirectly influences sheep oocyte development competence is an important goal. MT also significantly promoted the development of embryos *in vitro*, for example, in mice (31), bovines (32), swine (33), and goats (34).

### Direct Exogenous MT Employed in *in vitro* Culture

The beneficial effects of MT on early embryos depend on two key factors. An important function of it used *in vitro* is antioxidation. As an effective antioxidant, MT can significantly reduce the level of intracellular ROS and improve the *in vitro* maturation (IVM) of oocytes and the *in vitro* developmental potential of embryos (32). MT can significantly reduce the apoptosis of cultured cells and improve the quality of embryos. Previous studies have confirmed that MT can inhibit apoptosis and decreases in cell proliferation of sheep granulosa cells under heat stress (35). MT can inhibit the expression of the apoptosis genes *p53* and *Bax* and promote the expression of antioxidant genes *SOD1* and *GPX4* in embryos (36). In addition to its antioxidant properties, MT activates the SIRT1/PGC-1α pathway to compensate for mitochondrial depletion and energy deficiency caused by environmental toxin exposure and thereby promotes mitochondrial biosynthesis (33). MT can activate two G protein coupled receptors, MT1 and MT2, which regulate the growth of oocytes (37, 38). In addition, the effect of MT on oocytes may also be related to apoptosis, mitochondrial function (39), antioxidant enzymes (40), DNA methylation (41), cumulus cell expansion (42), and histone acetylation (43).

Our results show that MT is positively related to follicle growth *in vitro*. When cumulus oocyte complexes (COCs) were cultured alone, MT was positively related to IVM and blastocyst rate. That does not affect the quality of blastocysts, because blastocyst cell number is generally used as the standard to measure the quality of zygotes.

### Implanting MT in Ewes/Rams: Indirect Effects

Sheep are seasonal estrous animals, and the Assaf sheep are more seasonal than Merino sheep (9). MT is the key endogenic hormone regulating the estrus of sheep and that secretion is affected by light and by the season (44, 45). It can be secreted by cells of all tissues (1). MT is lipophilic and can penetrate all cell membranes and enter all tissues (38) and is particularly concentrated in follicles (46). The concentration of MT in follicles varies according to follicle size (2). The accumulation of ROS in the ovary will reduce the quality of oocytes and lead to the apoptosis of granulosa cells (47). MT can significantly reduce the level of intracellular ROS (32). It can penetrate the blood-testis barrier (48). When it was implanted in rams, the concentration of MT in seminal vesicles increased (49), and implanted MT affected semen quality. Oral administration of MT can also improve the human fertilization rate (50) and increase the number of high-quality embryos (51). Differences of implanted MT can cause differences in the release rate. One study found that the concentration of MT in seminal plasma peaked after 7 days and then decreased (52). Another study showed that the concentration of MT in seminal plasma gradually increased, reached the highest level after 90 days, and then decreased (53). In addition, the peak time of MT concentration differed according to different implant seasons (54). About 90% of MT is degraded through the liver (55).

Our results show that sheep from different regions and varieties can be made to enter estrus by implanting MT. It is positively related to litter size. Interestingly, MT has clear effects *in vitro* culture sheep oocytes and improves the blastocyst rate, although the embryo quality is unaffected in lab experimentation. Embryo quality is determined by the number of blastocyst cells number. MT has no effect on the cleavage rate *in vivo* but improves the rate *in vitro*. That declare the direct and indirect effects of MT on sheep oocytes are completely different.

Other studies have shown that there are MT receptors MT1 and MT2 in the sheep oviduct, and MT1 and MT2 are regulated by E2 (56). Cleavage occurs in the oviduct, and thus, the hypothesis is that implanted MT could increase the concentration of MT in oviduct fluid. During the present study, we noted that *in vitro* studies observed that the effect on the cleavage rate occurred 48 h after fertilization. The *in vivo* studies observed that most of the cleavage rate effect occurred 24–36 h after fertilization. In this way, different key time points can distinguish early cleavage rate and total cleavage rate (57). Hence the hypothesis that MT can improve the early cleavage rate, while the total cleavage rate will not be affected.

### Proposed Relation of MT to Sheep Oocyte Developmental Competence

**Figure 4:**
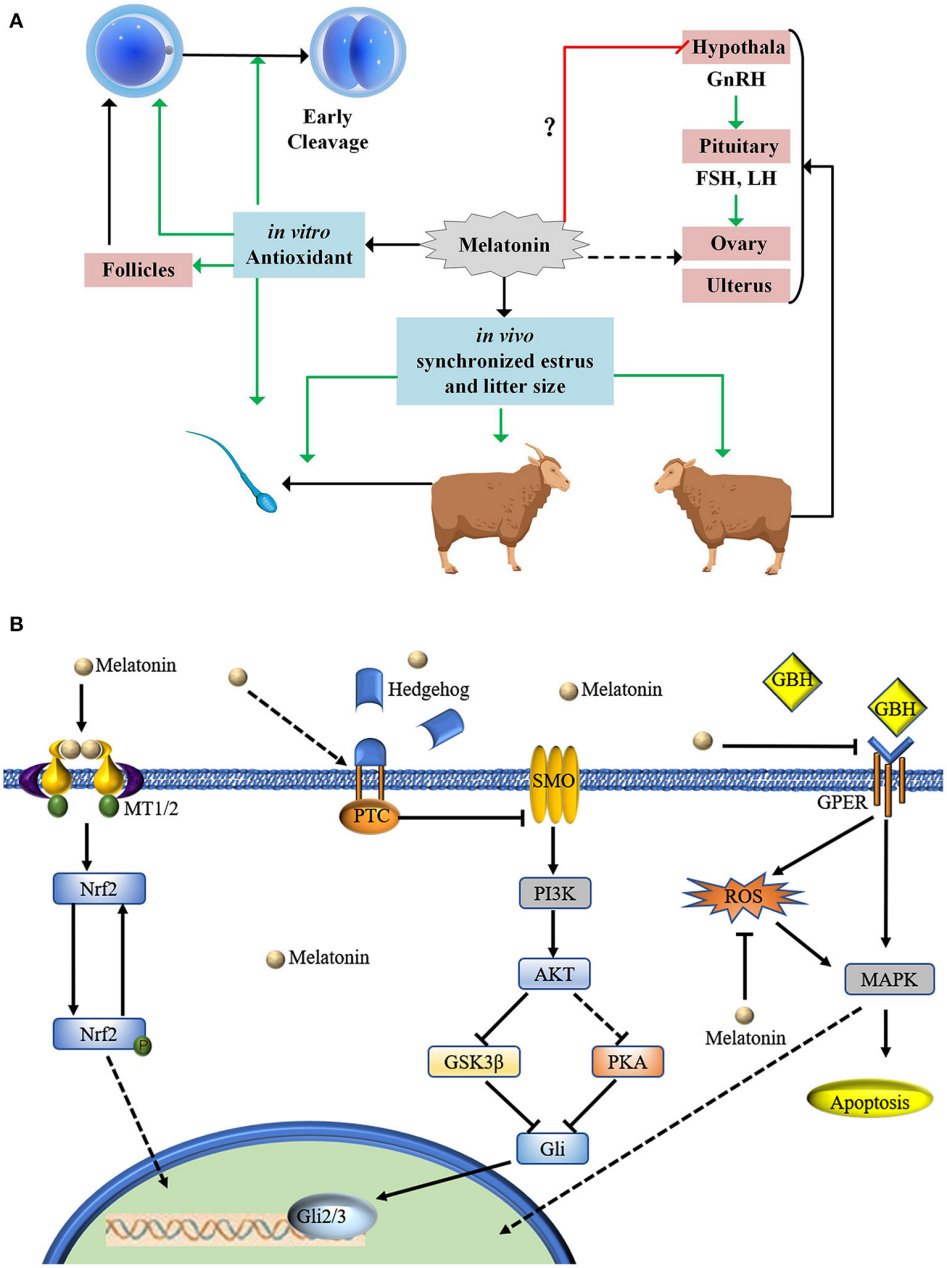
Proposed mode of action of MT in relation to sheep oocyte developmental competence. **(A)** MT treatment of sheep oocytes cultured *in vitro* has antioxidant effects and can increase oocyte development potential. MT can improve early cleavage. Implantation of MT in ewes and rams can promote estrus. There is no evidence to indicate whether implanting MT in ewes inhibits the hypothalamus-gonadal axis. Implanted MT can be enriched in follicles through the blood ovum barrier. Our statistical results show that implanting MT can increase the number of fetuses. **(B)** MT is involved in many oocyte-related signaling pathways.

MT is produced by the pineal gland. The normal secretion is a necessary condition for regulating physiological functions. However, excessive MT carries potential risks (58, 59). MT inhibits rat testosterone secretion by downregulating the hypothalamus-pituitary-gonadal axis (60). No studies have determined whether exogenic MT inhibits normal pituitary function (Figure 4A). MT is known to be involved in many oocyte-related signaling pathways. It is involved in the Hedgehog signaling pathway to promote swing oocyte IVM (61). MT reduces glyphosate herbicide toxicity during mouse oocyte maturation by regulating the GPER signaling pathway (62). MT-adjusted Nrf2 signaling decreased ROS in COCs and promoted swine (63) and mouse (64) oocyte IVM (Figure 4B). MT inhibited nicotinamide methylation signaling, promoting bovine oocyte IVM (65). In addition, MT is also involved in the Notch signaling pathway (66), the H4K16 deacetylation pathway (43), and the mTOR pathway (67).

Melatonin promotes the excretion of exosomes (68), and it promotes exosome secretion from mesenchymal stem cells also (69). Early embryonic blastomeres are totipotent stem cells (70). The concentration of MT differs in follicles according to follicle diameter (2). One aspect that needs to be considered in this area is how MT is enriched from the blood and how it can pass through the blood ovum barrier. It is involved in the cell crosstalk of oocytes and cumulus cells (71). If MT is secreted by granulosa cells or cumulus cells, this suggests that we should study the effect of it on oocytes from the aspects of endogenous MT autocrine and paracrine functions. MT can inhibit oocyte apoptosis (71). Regarding studies on oocyte apoptosis caused by injury (72, 73), we suggest that this should be confirmed by pyroptosis.

### Implication of This Research

Melatonin's effect on sheep oocyte development *in vitro* is conducive to the study of the underlying molecular mechanism, because itself is a hormone secreted by the body. The MT extracted from sheep is best used in the research process. Future research will focus on the mechanism of the interaction between MT and other hormones, the signaling pathways in which MT participates, and how it is transported across membranes against a concentration gradient.

Implanting MT to promote estrus is no longer the most suitable method of estrus regulation. The sheep and goats of our farms all use the method of vaginal sponges impregnated with progesterone for estrus synchronization, and then use laparoscope minimally invasive surgery to inject semen into the oviduct; this method has achieved good results.

Future studies need to determine whether MT can improve the early cleavage rate of sheep embryos without affecting the total cleavage rate. It is also necessary to determine whether implanting MT can increase the MT level in sheep oviduct fluid. Implanting MT affects all body organs and produces side effects such as promoting the growth of goat hair (74). It also affects the cardiovascular system (75), the neurological system (76), the endocrine system (77), and metabolism (78). Thus, implanting MT in non-breeding seasons should consider the effects on animal welfare.

## Data Availability Statement

The original contributions presented in the study are included in the article/Supplementary Material, further inquiries can be directed to the corresponding author/s.

## Author Contributions

YC and ZG collected the data and conducted the analysis. YC, XS, and ZG checked the data. HJ and ZG designed the experiment. All authors contributed to the article and approved the submitted version.

## Funding

This work was supported by the Jilin Scientific and Technological Developing Program (20200402039NC). National Key Research and Development Project of China (2021YFF1000702). The funders had no role in study design or preparation of the manuscript.

## Conflict of Interest

The authors declare that the research was conducted in the absence of any commercial or financial relationships that could be construed as a potential conflict of interest.

## Publisher's Note

All claims expressed in this article are solely those of the authors and do not necessarily represent those of their affiliated organizations, or those of the publisher, the editors and the reviewers. Any product that may be evaluated in this article, or claim that may be made by its manufacturer, is not guaranteed or endorsed by the publisher.
