# Supplementary material for: Exogenous Melatonin Directly and Indirectly Influences Sheep Oocytes
Source: Front Vet Sci. 2022 May 26;9:903195. doi: 10.3389/fvets.2022.903195 (PMC9203153; doi:10.3389/fvets.2022.903195)
Supplement: Supplementary file 1 [file Table_1.docx]

**Supplementary Data**

Supplementary data 1. Method of estimating the standard deviation for litter size.

Example: In Mura 2017, with 400 experimental sheep, the litter size was 1.2. Based on this, the original data were inferred. Because litter size can only be 0, 1, 2, or 3, and the probability of 0 and 3 is very low, this litter size can be ignored. In the original data, 320 ewes produced 1 lamb, and 80 ewes produced 2 lambs.

Average litter size was calculated as follows:

$$\bar{litter size}=\left( \sum_{i=1}^{80} 2+\sum_{i=1}^{320} 1 \right)\times\frac{1}{400}=1.2.$$

Standard deviation (SD) calculation:

$$SD=\sqrt[2]{\frac{\sum_{i=1}^{80} {(2-1.2)}^{2}+\sum_{i=1}^{320} {(1-1.2)}^{2}}{400-1}},$$

SD=0.4.
